# An Interpretable Hand-Crafted Feature-Based Model for Atrial Fibrillation Detection

**DOI:** 10.3389/fphys.2021.657304

**Published:** 2021-05-13

**Authors:** Rahimeh Rouhi, Marianne Clausel, Julien Oster, Fabien Lauer

**Affiliations:** ^1^Université de Lorraine, CNRS, LORIA, Nancy, France; ^2^Université de Lorraine, CNRS, IECL, Nancy, France; ^3^IADI U1254, Inserm and Université de Lorraine, Nancy, France

**Keywords:** atrial fibrillation, feature importance, interpretability, feature selection, classification, computer-aided diagnosis

## Abstract

Atrial Fibrillation (AF) is the most common type of cardiac arrhythmia. Early diagnosis of AF helps to improve therapy and prognosis. Machine Learning (ML) has been successfully applied to improve the effectiveness of Computer-Aided Diagnosis (CADx) systems for AF detection. Presenting an explanation for the decision made by an ML model is considerable from the cardiologists' point of view, which decreases the complexity of the ML model and can provide tangible information in their diagnosis. In this paper, a range of explanation techniques is applied to hand-crafted features based ML models for heart rhythm classification. We validate the impact of the techniques by applying feature selection and classification to the 2017 CinC/PhysioNet challenge dataset. The results show the effectiveness and efficiency of SHapley Additive exPlanations (SHAP) technique along with Random Forest (RF) for the classification of the Electrocardiogram (ECG) signals for AF detection with a mean F-score of 0.746 compared to 0.706 for a technique based on the same features based on a cascaded SVM approach. The study also highlights how this interpretable hand-crafted feature-based model can provide cardiologists with a more compact set of features and tangible information in their diagnosis.

## 1. Introduction

Atrial Fibrillation (AF) is the most common cardiac arrhythmia with an increased prevalence with aging (Chugh et al., 2014). AF is defined by an irregular Heart Rate (HR), caused by a chaotic electrical activity in the atria. It can lead to the formation of clots, heart failure, and other heart-related abnormalities (Wolf et al., 1991), and is associated with a five-fold increased risk of stroke (Wolf et al., 1978). The approximated direct costs spent for AF is about 1% of the total healthcare expenditure in the UK and about 6-26 billion US dollars in the US in 2008 (Stewart et al., 2004).

The Electrocardiogram (ECG) has been extensively investigated for the diagnosis of many cardiac diseases. In a Computer-Aided Diagnosis (CADx) system for heart rhythm classification, features are extracted from an ECG signal and are a (compact) representation of the corresponding signal, which are fed into a Machine Learning (ML) model. ML models automatically learn useful patterns from training data (including the extracted features from the ECG signals) for which the diagnosis is already known and aim at extracting knowledge into their structures and parameters. The development of automated AF detection has attracted an increased level of attention, since the combination of wearable devices and ML has been seen as a potential solution for an early management of AF in order to prevent adverse events such as stroke.

During the last decade, there has been an explosion of AF detection algorithms. Interested readers are referred to Sörnmo (2018). The availability of open-source ECG databases on the PhysioNet website and through recent CinC/PhysioNet challenges has allowed for the development of novel ML techniques, among which most recently deep learning (DL) approaches. Automated AF detection can be divided into three categories (i) classical ML classifiers using specifically hand-crafted features extracted from the ECG signals (ii) fully automated DL approaches based on Convolutional Neural Network (CNN), Recurrent Neural Networks (RNN) or Transformers, or (iii) hybrid approaches using a combination of hand-crafted features and DL techniques. Hand-crafted features are designed in order to extract rhythm-based information (and the irregularity of RR intervals) (Sarkar et al., 2008; Bashar et al., 2020; Lown et al., 2020), or morphological features (detection of the absence of P-wave and presence of f-waves) or both rhythm and morphology features (Behar et al., 2017; Datta et al., 2017; Zabihi et al., 2017; Sörnmo, 2018). Many DL approaches have been suggested either by applying 1d-CNN to single lead ECG directly (Pyakillya et al., 2017), or by transforming the ECG signals into an image through a time-frequency transform such as wavelet transform (He et al., 2018), or by adding an RNN layer after the CNN (Warrick and Homsi, 2018). Finally, hybrid approaches have also been suggested with the combination of automatically extracted features with CNN and hand-crafted features (Liaqat et al., 2020). Teijeiro et al. (2018) suggested the use of hand-crafted features and RNN for temporal analysis of ECG signals, and obtained excellent results on the 2017 CinC/PhysioNet challenge.

ML models can often be so-called black boxes, whose internal logic and inner functionality are hidden, preventing them from easily verifying, interpreting, and understanding the reasoning of the system and how particular decisions were taken. For clinical applications and to gain the trust of end-users (clinicians), it is crucial to be able to explain model predictions and provide cardiologists with tangible information explaining why a given prediction was made.

As a prevailing solution to the explanation issue, feature importance techniques indicate the contribution of each feature to the output. A first approach consists in using so-called *interpretable* models such as decision trees (Breiman et al., 1984), additive models (Caruana et al., 2015), attention-based networks (Xu et al., 2015), or sparse linear models (Ustun and Rudin, 2016). In these approaches, one uses models in which there is the possibility of meaningfully investigating model components directly, e.g., considering a path in a decision tree, or the weight of a specific feature in a linear model. As long as the model is accurate for the task, and uses a reasonably restricted number of internal components (i.e., paths, rules, or features), such approaches provide extremely useful insights.

The situation is much more complex when we have to extract explanations from a black-box model. To tackle this setting, several strategies can be developed. One can either use a two-step procedure, based on distillation approaches (Hinton et al., 2015), learning at first an interpretable model on the predictions of the black box model and thereafter computing the feature importance for the white box model (Craven and Shavlik, 1996; Baehrens et al., 2010). In this paper, we shall focus on one-step-procedures, based on sensitivity analysis and its extensions (Christopher Frey and Patil, 2002; Iooss and Lemaître, 2015), where the feature importance is directly computed from the black-box model perturbing inputs and seeing how the black box model reacts (Strumbelj and Kononenko, 2010; Krause et al., 2016) or both (Ribeiro et al., 2016).

Generally, feature importance techniques are divided into either global or local explanation approaches. Global explanation focuses on feature-level importance scores for how much a given input feature contributes to a model output (Bhatt et al., 2020). Local explanation focuses on the contribution of features for a specific observation (i.e., for a specific ECG record) (Murtaza et al., 2020).

In this paper, we present a range of interpretability techniques applied to hand-crafted features based machine learning models for heart rhythm classification. The objective is two-fold: (i) applying feature importance techniques in order to reduce the complexity of the ML classifier and providing a global explanation of the decision making process to the cardiologists (end-user), and (ii) providing local explanations of the decision making process to the end-user. It should be mentioned that the aim of this paper is not presenting the best model for AF classification but to highlight the benefits of interpretability for building a more compact set of features to provide cardiologists with tangible information in the classification. Accordingly, we introduce an interpretable hand-crafted feature-based model for AF classification.

The rest of the paper is organized as follows. In section 2, one first presents the data of interest and the machine learning task what we are performing on these data. Thereafter, in section 3 one reviews the main global and local explanation techniques for hand-crafted feature-based models. In section 4, results of the feature importance techniques and evaluation of the performance and the strength of each technique by feature selection and classification on CinC/PhysioNet 2017 dataset are presented. Also, an interpretable model for the classification is introduced. In section 5, the significance and limitations of the proposed methods are discussed in details. Finally, conclusion is given in section 6.

## 2. The Rhythm Classification Task

In this section, one first describes the CinC/Physionet dataset as well as a succinct list of features extracted and the rhythm classification task. Thereafter, one introduces the different classifiers tested and the quality assessment technique.

### 2.1. Dataset and Feature Extraction

This study focused on the analysis of the dataset from the 2017 PhysioNet/Computing in Cardiology (CinC) challenge (Clifford et al., 2017), collected using a mobile health device (the AliveCor device), as it constitutes one of the largest dataset of single lead ECG with heart rhythm annotations. The dataset includes 8528 single lead ECG signals between 9 and 60 s in length, which were sampled at 300 Hz and filtered by a band pass filter. The signals were labeled into four classes: atrial fibrillation (A) (735 samples), normal sinus rhythm (N) (5,050 samples), other rhythms (O) (2,456 samples), and noisy recordings (~) (284 samples).

A set of 56 features are extracted from each individual signal in the dataset based on Behar et al. (2017). These hand-crafted features were designed for different purposes: (i) assessing the quality of the recording (ii) analyzing the morphology of the ECG (either measuring the QRS width, detecting P waves or assessing the presence of f-waves) and (iii) analyzing the regularity of the RR intervals [either standard HR variability (HRV) measures or specific measures suggested for the detection of AF (Coefficient of sample entropy and Poincare plot)]. The extracted features are listed in Table 1.

**Table 1:** List of features extracted from ECG signals.

**Index**	**Feature name**	**Description**
1	bSQI	Signal quality of the overall recording (Behar et al., 2013)
2	meanSQI	Mean Signal Quality Index (SQI) value over selected segment
3	medSQI	Median SQI value over selected segment
4	quarSQI	First quartile of SQI values over selected segment
5	len_seg	Length of selected segment
6	distQRS	Mean distance between the two QRS detcetors
7	CosEn	Coefficient of sample entropy (Lake and Moorman, 2011)
8	AFE	AFEvidence (Sarkar et al., 2008)
9	OrC	Number of points in the bin containing the Origin (Sarkar et al., 2008)
10	IrE	Irregularity Evidence (Sarkar et al., 2008)
11	PACe	Premature Atrial Contractions (PAC) Evidence (Sarkar et al., 2008)
12	al_rr	Ratio of RR intervals with alternating length
13	lv_rr	Ratio of RR intervals with large variations
14	bi_rr	Bimodal RR interval distribution
15	min_rr	Minimum RR interval
16	max_rr	Maximum RR interval
17	med_rr	Median RR interval
18	nb_out	RR-interval outliers. An outlier was defined as a sample exceeding 20% of a window average of size 12 beats
19	HR	Type of heart rate irregularity, tachycardia or bradycardia
20	medQS	Median QRS width
21	stdQS	Standard deviation of the QRS width
22	medR	Median R-peak amplitude (mV)
23	stdR	Standard deviation of the R-peak amplitude (mV)
24	Ratio	Ratio of the power spectral frequency in the band 5-9 Hz normalized by the total power frequency computed on the PQRST canceled signal
25	max_freq	Peak frequency in the band 4-45 Hz from the power spectrum computed on the PQRST canceled signal
26	medQT	Median distance from Q_on_ to T_off_
27	medQT_b	Median QT interval corrected using Bazett's formula
28	medQT_fre	Median QT interval corrected using Frederica's formula
29	medQT_fra	Median QT interval corrected using Framingham's formula
30	medQT_hod	Median QT interval corrected using Hodges' formula
31	medP	Median P-wave length defined as the distance from P_on_ to P_off_
32	med*PR*	Median PR interval defined as the distance from P_on_ to Q_on_
33	stdPR	Standard deviation of the PR interval
34	medT	Median T-wave length defined as the distance from T_on_ to T_off_
35	medTamp	Median T amplitude computed as the amplitude in mV between the T_off_ to the peak of the T-wave
36	stdTamp	Standard deviation of the T-wave amplitude
37	Ttype	Type of T-wave morphology (normal, inverted, and …)
38	stdP	Standard deviation of the P-wave length
39	stdT	Standard deviation of the T-wave length
40	PIP	Percentage of inflection points (%) (Costa et al., 2017; Rosenberg, 2017)
41	IALS	Inverse average length of segments (Costa et al., 2017; Rosenberg, 2017)
42	PSS	Percentage of NN intervals that are in short segments (Costa et al., 2017; Rosenberg, 2017)
43	AVNN	Average NN interval duration (ms) (Rosenberg, 2017)
44	SDNN	Standard deviation of NN interval duration (ms) (Rosenberg, 2017)
45	RMSSD	Root-mean-squared difference between adjacent NN intervals (ms) (Rosenberg, 2017)
46	pNN50	Percent of NN interval differences greater than 50 ms (%) (Rosenberg, 2017)
47	SEM	Standard error of the mean NN interval (ms) (Costa et al., 2017; Rosenberg, 2017)
48	PAS	Percentage of NN intervals that are in alternation segments of at least 4 intervals(%) (Costa et al., 2017; Rosenberg, 2017)
49	nbpwaves	Number of P-waves detected by cardiac cycles
50	medPamp	Median P-wave amplitude defined as the amplitude of the P-wave computed from P_off_ to the peak of the P-wave
51	stdPamp	standard deviation P-wave amplitude defined as the amplitude of the P-wave computed from P_off_ to the peak of the P-wave
52	med_tb	Binary test for tachycardia or bradycardia
53	medSTvar1	Amplitude of the ST segment measured at J-Point
54	medSTvar2	Amplitude of the ST segment measured at J-Point + 60 ms
55	medST	Median segment length defined as the distance between QRS_off_ and T_on_
56	medPRseg	Median PR segment defined as the distance from P_off_ to Q_on_

### 2.2. Supervised ML Approaches for Rhythm Classification

We try with different supervised classifiers such as Support Vector Machine (SVM) (Cortes and Vapnik, 1995), Logistic Regression (LR) (Hosmer et al., 2013), Random Forest (RF) (Breiman, 2001) and Gradient Boosting (GB) (Friedman, 2001). Each classifier is trained on the training set and tested on the test set, including the extracted features from ECG signals and their corresponding labels. We also apply a cascaded form of the mentioned classifiers. It could be a way to handle the imbalanced dataset CinC/PhysioNet in which the class N samples are almost two-third of all the recordings (Behar et al., 2017). Hence, we try with the cascaded form of the classifiers which are Cascaded SVM (CSVM), Cascaded LR (CLR), Cascaded RF (CRF), and Cascaded GB (CGB). More specifically, in the cascaded classification, regarding the applied dataset, including four classes, three binary classifiers are created. The first one classifies samples into two classes, i.e., the class N and the rest. The second one classifies samples into two classes A and the rest. The third one classifies samples into the two classes O and ~.

### 2.3. Quality Assessment

The effectiveness of a classifier can be assessed by computing the number of correctly recognized class samples, i.e., True Positives (TP), the number of correctly recognized samples that do not belong to the class, i.e., True Negatives (TN), and samples that either were incorrectly assigned to the class, i.e., False Positives (FP), or that were not recognized as class samples, i.e., False Negatives (FN) (Sokolova and Lapalme, 2009). For multi-class problems with *l* categories, the validation is defined, for each individual class *C*_*i*_, by *TP*_*i*_, *FN*_*i*_, *TN*_*i*_, and *FP*_*i*_. The quality of the classification can be assessed in two ways: the sum of counts to obtain cumulative *TP*, *FN*, *TN*, *FP* and then calculating a measure (micro-averaging shown with the μ index), or the average of the same measures calculated for *C*_1_, …, *C*_*l*_ (macro-averaging shown with the M index). Macro-averaging treats all classes equally, while micro-averaging favors bigger classes. Accordingly, precision (P), recall (R), and F-score (F) are defined as follows (Rijsbergen, 1979):

(1) $$\begin{array}{l} P_{\mu }=\frac{\sum_{i=1}^{l}{TP}_{i}}{\sum_{i=1}^{l}\left({TP}_{i}+{FP}_{i}\right)} \end{array}$$

(2) $$\begin{array}{l} R_{\mu }=\frac{\sum_{i=1}^{l}{TP}_{i}}{\sum_{i=1}^{l}\left({TP}_{i}+{FN}_{i}\right)} \end{array}$$

(3) $$\begin{array}{l} F_{\mu }=\frac{2\;P_{\mu }R_{\mu }}{P_{\mu }+R_{\mu }} \end{array}$$

(4) $$\begin{array}{l} P_{M}=\frac{\sum_{n=1}^{l}\frac{{TP}_{i}}{{TP}_{i}+{FP}_{i}}}{l} \end{array}$$

(5) $$\begin{array}{l} R_{M}=\frac{\sum_{n=1}^{l}\frac{{TP}_{i}}{{TP}_{i}+{FN}_{i}}}{l} \end{array}$$

(6) $$\begin{array}{l} F_{M}=\frac{2\;P_{M}R_{M}}{P_{M}+R_{M}} \end{array}$$

It should be noted that the values of $P_{\mu }$, $R_{\mu }$ and $F_{\mu }$ are equal (Sokolova and Lapalme, 2009). Hence, in the results, we only report $F_{\mu }$. To assess the classification generalization, we use repeated 10-fold cross validation. In 10-fold cross validation, the dataset is divided into 10- and 9-folds are considered for training and one-fold is considered for the test (Stone, 1974). Then, the average of values of each measure for the test folds is considered for the evaluation of the classifier. Besides, we apply the non-parametric statistical Wilcoxon test (Cuzick, 1985) to assess the statistically significant difference between methods. We also use Receiver Operating Characteristic (ROC) (Fawcett, 2006) and Area Under the ROC curve (AUC) for the evaluation of classification. ROC is plotted in a two-dimensional space in which the x-axis and y-axis represent the True Positive Rate (TPR) or R and the False Positive Rate (FPR), respectively (Sokolova and Lapalme, 2009).

## 3. Feature Importance

### 3.1. Global Explanation and Feature Selection

Global explanation aims to provide an understanding on ML models and highlight the most important parameters or learned representations along with an explanation of these features in an intuitive manner to end-users. Global explanation techniques are trained on the overall training set and provide therefore an overall perspective for a model. These techniques aim to answer to *how does the trained model make predictions?* The next sections will give a description of the global explanation techniques used in this paper while the last subsection will introduce how these techniques can be validated as a feature selection approach.

#### 3.1.1. Logistic Regression (LR)

LR is an extension of the linear regression which models the relationship between a categorical response variable *y* and a set of *x* ∈ ℝ^*k*^ of *k* explanatory variables, by fitting a linear equation (Tolles and Meurer, 2016). Given a training set (*x*_*i*_, *y*_*i*_), *i* = 1, …, *n*, the goal is to find the LR function *p*(*x*) so the responses *p*(*x*_*i*_) as closely as possible the actual response *y*_*i*_ for each observation *x*_*i*_, where *i* = 1, 2, …, *n*. In a binary LR, probabilities are modeled with two possible outcomes, meaning that *p*(*x*_*i*_) should be equal to either 0 or 1. A linear function *f*(*x*) = *b*_0_ + *b*_1_*x*_1_ + ... + *b*_*k*_*x*_*k*_, is applied, where the variables *b*_0_, *b*_1_, …, *b*_*k*_ are the estimators of the regression coefficients, so-called the predicted weights or coefficients. The model coefficients can be interpreted as indicators of feature importance. These coefficients can provide the basis for a crude feature importance score, assuming that the input features have the same scale or have been scaled prior to fitting a model. The LR function *p*(*x*) is the sigmoid function of *f*(*x*) calculated by

(7) $$\begin{array}{l} p\left(x\right)=\frac{1}{1+exp\left(-f\left(x\right)\right)} \end{array}$$

LR estimates the coefficients *b*_0_, *b*_1_, ..., *b*_*k*_ such that the function *p*(*x*) best approximates all actual responses *y*_*i*_ ∈ {0, 1}, *i* = 1, ..., *n*. During the training step, the Log-Likelihood Function (LLF) (Minka, 2001) for all samples, defined as

(8) $$\begin{array}{l} \text{LLF}=\overset{n}{\underset{i=1}{\sum }}\left(y_{i}\log\left(p\left(x_{i}\right)\right)+\left(1-y_{i}\right)\log\left(1-p\left(x_{i}\right)\right)\right), \end{array}$$

is maximized.

LR is easily implemented, and results in a good accuracy for many simple datasets and performs ideally when the dataset is linearly separable, but is not flexible enough to fit complex datasets and it can overfit in high-dimensional datasets.

#### 3.1.2. Random Forest (RF)

Random Forest (RF) is an ensemble model including decision trees as base learners, each learning a different aspect of data and class prediction. The class with the most votes becomes the RF's prediction. RF considers a random subset of features for making the trees. Considering a node τ within the binary tree *T* in the RF, the optimal split is obtained by the *Gini impurity* measure (Breiman, 2001) denoted by *G*(τ). Gini impurity is a computationally efficient approximation of the entropy measuring how well a potential node splits the samples of the two classes. With $p_{k}=\frac{n_{k}}{n}$ being the fraction of the *n*_*k*_ samples from the category *k* ∈ {0, 1} out of the total of *n* samples at node τ, the Gini impurity *G*(τ) is calculated as follows:

(9) $$\begin{array}{l} G\left(\tau \right)=1-p_{1}^{2}-p_{0}^{2} \end{array}$$

The decrease of *G*(τ), specified by Δ*G*, resulting from a split and the division of the samples into two sub-nodes τ_*l*_ and τ_*r*_ with related sample fractions $p_{l}=\frac{n_{l}}{n}$ and $p_{r}=\frac{n_{r}}{n}$, according to a threshold *t*_θ_ on feature θ, is defined as follows:

(10) $$\begin{array}{l} \Delta G\left(\tau \right)=G\left(\tau \right)-p_{l}G\left(\tau_{l}\right)-p_{r}G\left(\tau_{r}\right) \end{array}$$

In an exhaustive search over all features θ available at the node τ, a property of the RF is to restrict this search to a random subset of the available features (Breiman, 2001), and over all possible thresholds *t*_θ_, the pair {θ, *t*_θ_} leading to a maximal Δ*G* is determined. For any feature θ, the decrease in Gini impurity resulting from this optimal split, Δ*G*_θ_(τ, *T*), is stored and accumulated for all nodes τ in all trees *T* in the forest, in the Gini importance

(11) $$\begin{array}{l} I_{G}\left(\theta \right)=\underset{T}{\sum }\underset{\tau }{\sum }\Delta G_{\theta }\left(\tau ,T\right) \end{array}$$

The Gini importance *I*_*G*_ indicates how often a particular feature θ is selected for a split, and how discriminating it is for the classification. The Gini importance values can be used as values of feature importance (Guyon and Elisseeff, 2003). The advantage of RF is that, unlike LR, it requires no prior knowledge on the linear separability of the classes. The learning is agnostic and it is much more general and applicable to even large datasets.

#### 3.1.3. Permutation Testing (PT)

Permutation Testing (PT) estimates the importance of a particular feature based on the overall results of an underlying machine learning model (Breiman, 2001). It applies permutations to features and re-calculate the prediction accuracy. The feature importance is defined as the mean decrease in the accuracy of the trained model when each feature is permuted. Especially, Breiman (2001) proposed measuring the importance of the *j*^*th*^ feature by permuting its values in the training data and examining the corresponding drop in predictive accuracy on a model built with the original training data. Given a training set consisting of a data matrix

(12) $$\begin{array}{l} X=\left[\begin{array}{c} x_{1}^{⊤}\\ \vdots \\ x_{n}^{⊤} \end{array}\right]=\left[\begin{array}{ccc} x_{11} & \cdots & x_{1k}\\ \vdots & \ddots & \vdots \\ x_{n1} & \cdots & x_{nk} \end{array}\right] \end{array}$$

and corresponding response vector $y={\left[y_{1},y_{2},...,y_{n}\right]}^{⊤}$, let *X*^π,*j*^ be a matrix obtained by randomly permuting the entries in the *j*^*th*^ column of *X* containing the values of the *j*^*th*^ feature for all the samples *x*_*i*_, *i* = 1, ..., *n*. Using *L*(*y*_*i*_, *f*(*x*_*i*_)) as the loss for predicting *f*(*x*_*i*_) instead of *y*_*i*_ (Breiman, 2001) determined the importance of the *j*^*th*^ feature as

(13) $$\begin{array}{l} PI_{j}^{\pi }=\overset{n}{\underset{i=1}{\sum }}L\left(y_{i},f\left(x_{i}^{\pi ,j}\right)\right)-L\left(y_{i},f\left(x_{i}\right)\right) \end{array}$$

the increase in loss which is due to replacing *x*_*ij*_ with a value randomly chosen from the (marginal) distribution of feature *j*. The authors of Breiman (2001) designed the method specifically with the RF as the underlying model and considered OOB loss, based only on trees that were not trained using (*x*_*i*_, *y*_*i*_). For more general learners, either training or test loss can be used. The main advantage of such a PT approach is that it is scalable for any model. Most studies using the related permutation-based feature importance of RFs (Díaz-Uriarte and De Andres, 2006; Shen et al., 2007) together with RFs in a recursive feature elimination scheme, also showed an increase in prediction performance. Only Li et al. (2005) report a constant performance, but with a greatly reduced amount of features. Permutation importance also allows us to make “apples-to-apples” comparisons of the importance of different models trained on the same data. Disadvantages of PT include its complexity and its inability to handle feature interactions. Permutation importance scores require generating predictions on the test set twice for each feature, which may be computationally intractable for large feature spaces. The permutation scores also do not consider those predictors may naturally vary together. This can cause misleading interpretations for certain models (Strobl et al., 2008).

#### 3.1.4. SHapley Additive exPlanations (SHAP)

SHAP works based on the concept of Shapley value (Shapley, 1953) developed in cooperative game theory to estimate how much each player contributes in a coalition and receives a payout based on the contribution (Shapley, 1953). The aim of Shapley values is to find which player is the most important one in the cooperative game environment. Taking the idea into machine learning and interpretability context, the goal is to figure out which feature plays the most important role in a specific prediction. Correspondingly, here, the prediction task becomes a game, feature values are players and feature contributions are payouts. By applying game theory concepts, SHAP guarantees that there is a unique solution to a new class that helps to measure the unified SHAP values, approximated by various methods. SHAP represents an additive feature attribution method, which enables the connectivity of various explanation models, including LIME, within the SHAP framework. Additive feature attribution methods have an explanation model that is a linear function of binary variables:

(14) $$\begin{array}{l} \psi \left(z^{\prime }\right)=\phi_{0}+\overset{M}{\underset{i=1}{\sum }}\phi_{i}z_{i}^{\prime } \end{array}$$

where ψ is an interpretable model, *z*′ ∈ {0, 1}^*M*^ is a simplified feature vector where 0 denotes the absence of feature value and 1 denotes the presence. *M* is the number of simplified input features and ϕ_*i*_ ∈ ℝ is the feature attribution for feature *i*, i.e., the Shapley values. SHAP proposed a way to transform the underlying interpretable models into

(15) $$\begin{array}{l} f\left(x\right)=\psi \left(x^{\prime }\right)=\phi_{0}+\overset{M}{\underset{i=1}{\sum }}\phi_{i}x_{i}^{\prime } \end{array}$$

and then unifies explanation method who satisfies three desirable properties of Shapley values (Molnar, 2019). The first desirable property is local accuracy, and it measures how well an explanation method estimates the output of function *f*(*x*) for a simplified input *x*_0_, where *x*_0_ corresponds to an original sample *x* that is being explained and *f*(*x*) is a black-box model which predicts an output for *x*. In order to see if an explanation model ψ(*x*′) matches the original model *f*(*x*), a function $x=h_{x}\left(x^{\prime }\right)$ first transforms the simplified input *x*_0_ to original sample *x*. The second desirable property is missingness. It indicates that when $x_{j}^{\prime }=0$, then the feature should not have attribution impact, i.e., $x_{j}^{\prime }=0\Rightarrow \phi_{j}=0$. The third property is consistency. It states that if some changes in a model increase the input's contribution, it should not decrease the input's attribution. Let $f_{x}\left(z^{\prime }\right)=f\left(h_{x}\left(z^{\prime }\right)\right)$ and *z*′\*j* denote *z*′ with its *j*^*th*^ entry set to 0. For any two models *f* and *f*′, if $f_{x}^{\prime }\left(z^{\prime }\right)-f_{x}^{\prime }\left(z^{\prime }\backslash j\right)\geq f_{x}\left(z^{\prime }\right)-f_{x}\left(z^{\prime }\backslash j\right)$ for all inputs *z*′ ∈ {0, 1}^*M*^ if follows that $\phi_{j}\left(f^{\prime },x\right)\geq \phi_{j}\left(f,x\right)$. In the context of a Shapley value, it means that if model changes increase the marginal contribution of a feature value, or even the marginal contribution remains the same (regardless of the other features), then the Shapley value of the feature should not decrease, it should also increase or stays the same.

In our implementation, we use TreeExplainer (Lundberg et al., 2020) which is particularly relevant for explaining tree-based machine learning models like RF. TreeExplainer presents fast explanations of the model with guaranteed consistency. It provides the exact computation of Shapley values in low-order polynomial time by leveraging the internal structure of tree-based models. Shapley values need a summation of terms across all possible feature subsets. TreeExplainer falls this summation into a set of calculations specific to each leaf in a tree. This is an incremental improvement in terms of complexity over previous exact Shapley methods. Explanations based on TreeExplainer provide a global understanding of the model structure. The average Shapley value per feature across all instances can be considered as feature importance values. In this case, the importance value represents the extent to which the feature influences the outcome and not the model performance or model construction.

#### 3.1.5. Feature Selection

We validate the impact of the global feature importance techniques by feature selection and classification. As explained before, the output of each feature importance technique is a ranking list of features, specifying their importance in the heart rhythm classification. We use these rankings to select the most important features, to the extent they generate almost the same results once all the features are selected. For SHAP technique which provides separate importance values for each class, to get a general importance value, we average the resulted importance values for a given feature for all the classes.

### 3.2. Local Explanation

Contrary to global explanation techniques, local explanation tries to explain predictions on a single data-point and mainly addresses the question of *why did the model make a specific prediction?* This study focused on two local explanation techniques Local Interpretable Model-agnostic Explanations (LIME) and SHAP are explained as follows. In the following, two local explanation techniques are described.

#### 3.2.1. Local Interpretable Model-Agnostic Explanations (LIME)

Local Interpretable Model-agnostic Explanations (LIME) is an explanation technique that provides local explanations, in the sense that it yields explanations for each individual prediction (Ribeiro et al., 2016). Each part of the name reflects something that is desirable in explanations. “Local” refers to local fidelity, i.e., we want the explanation to really reflect the behavior of the classifier “around” the instance being predicted. Some classifiers use representations that are not intuitive to users at all (e.g., word embeddings). LIME explains those classifiers in terms of “interpretable” representations, even if that is not the representation actually used by the classifier. Further, LIME takes human limitations into account, i.e., the explanations are not too long. In order to be “model-agnostic,” LIME cannot peak into the model. To figure out what parts of the interpretable input are contributing to the prediction, the input around its neighborhood is perturbed to see how the model's predictions behave. Then, these perturbed data points are weighted by their proximity to the original example. The training set containing permuted samples and their related predictions by the model is applied to train and evaluate a local interpretable model (a linear model) and approximate the model in the vicinity of the sample being explained.

Let ψ ∈ Ψ be an explanation model where Ψ is a class of interpretable models such as linear models or decision trees in RF. As explanation should be simple enough to understand, so the domain of ψ is {0, 1}^*d*^′, which shows the absence or presence of the *d*′ components in its interpretable representation. The original representation of a sample being explained is *x* ∈ ℝ^*d*^, but to make an explanation interpretable, a binary vector representation *x*′ ∈ {0, 1}^*d*^′ is used as an interpretable representation. Besides, Ω(ψ) is used as a measure to control the complexity of an explanation model ψ. For example, in the case of linear models, the complexity can be the number of non-zero weights, while it can be the depth of the tree for decision trees. Let *f* : ℝ^*d*^ → ℝ depicts a model being explained and *f*(*x*) is a probability function that determines that sample *x* belongs to a certain class. To explain the prediction locally, π_*x*_(*z*) is used as a proximity measure between a sample *z* and *x* to define locality around *x*. In the original work of (Ribeiro et al., 2016), π_*x*_(*z*) is set to an exponential kernel exp(−*D*(*x, z*)^2^/σ^2^) defined on some distance measure *D* with width σ. The explanation can be obtained using

(16) $$\begin{array}{l} \xi \left(x\right)=\underset{\psi \in \text{Ψ}}{\arg\min}L\left(f,\psi ,\pi_{x}\right)+\Omega \left(\psi \right) \end{array}$$

where $L\left(f,\psi ,\pi_{x}\right)$ is a measure of how unfaithful ψ is in approximating *f* in the locality defined by π_*x*_. The goal is to minimize the $L\left(f,\psi ,\pi_{x}\right)$ while keeping Ω(ψ) small enough to produce an understandable explanation. LIME only implements a class of linear models Ψ as interpretable models $\psi \left(z^{\prime }\right)=w_{\psi }^{⊤}z^{\prime }$ and develops a linear model using sampled dataset Z. The dataset Z contains samples *z*′ ∈ {0, 1}^*d*^′ drawn uniformly at random from non-zero elements of *x*′, weighted by π_*x*_. The labels for the sampled instances *z*′ ∈ {0, 1}^*d*^′ are generated by using the main probability function *f*(*z*). The function requires an original representation *z* ∈ ℝ^*d*^ of a sample, which can be recovered from interpretable representation *z*′ ∈ {0, 1}^*d*^′. So, $L\left(f,\psi ,\pi_{x}\right)$ in (16) is defined as

(17) L(f,ψ,πx)=∑z,z′∈Zπx(z)(f(z)-ψ(z′))2

Finally, by using the dataset Z and the optimization in (16), the local explanation ξ(*x*) for the sample *x* is provided. As we deal with the extracted features from ECG signals and their corresponding labels, we apply the implementation of LIME for tabular data, through which new samples are created by perturbing each feature individually, drawing from a normal distribution with mean and standard deviation taken from the feature.

#### 3.2.2. SHapley Additive exPlanations (SHAP)

The baseline of the SHAP technique was presented in section 3.1.4.

Both local explanation techniques will be presented in the results section by depicting examples of output that could be provided along with the heart rhythm prediction to the cardiologists. They will therefore be provided with an explanation as to why the classifier decided to make its decision for a given ECG signal.

## 4. Results

The first subsection presents the results of the different classifiers. The second subsection shows how the global explanation is used in feature selection and impacts the results of the classification. Finally, several examples of results of both tested local feature importance techniques will be presented.

### 4.1. Classification

Different classifiers (i.e., SVM, LR, RF, GB and their cascaded form CSVM, CLR, CRF, and CGB) were applied and tested by inputting all 56 features implemented. The classification was evaluated using different measures defined in (1–6). The results presented in Table 2 show that the RF classifier achieved the best performance (the best value of each measure is highlighted in Bold). Hence, in the remaining of the document and for the following experiments, we applied an RF classifier.

**Table 2:** Results of different classifiers applied to 56 extracted features based on 10-fold cross validation.

**Classifier**	**$F_{\mu }$**	**$P_{M}$**	**$R_{M}$**	**$F_{M}$**
SVM	0.812^*^	0.780	0.697^*^	0.726^*^
CSVM	0.723^*^	0.745^*^	0.698	0.701^*^
LR	0.813^*^	0.770^*^	0.699	0.726^*^
CLR	0.738^*^	0.701^*^	0.702	0.726^*^
RF	**0.833**	**0.779**	0.713	**0.741**
CRF	0.776^*^	0.764^*^	0.727^*^	0.736^*^
GB	0.828^*^	0.776	0.706^*^	0.735^*^
CGB	0.740^*^	0.677^*^	0.682^*^	0.669^*^

(*) *shows the non-parametric statistical difference between RF classifier and the corresponding classifier in terms of a specific measure $F_{\mu }$, $P_{M}$, $R_{M}$, or $F_{M}$*.

### 4.2. Global Explanation and Feature Selection

**Figure 1:**
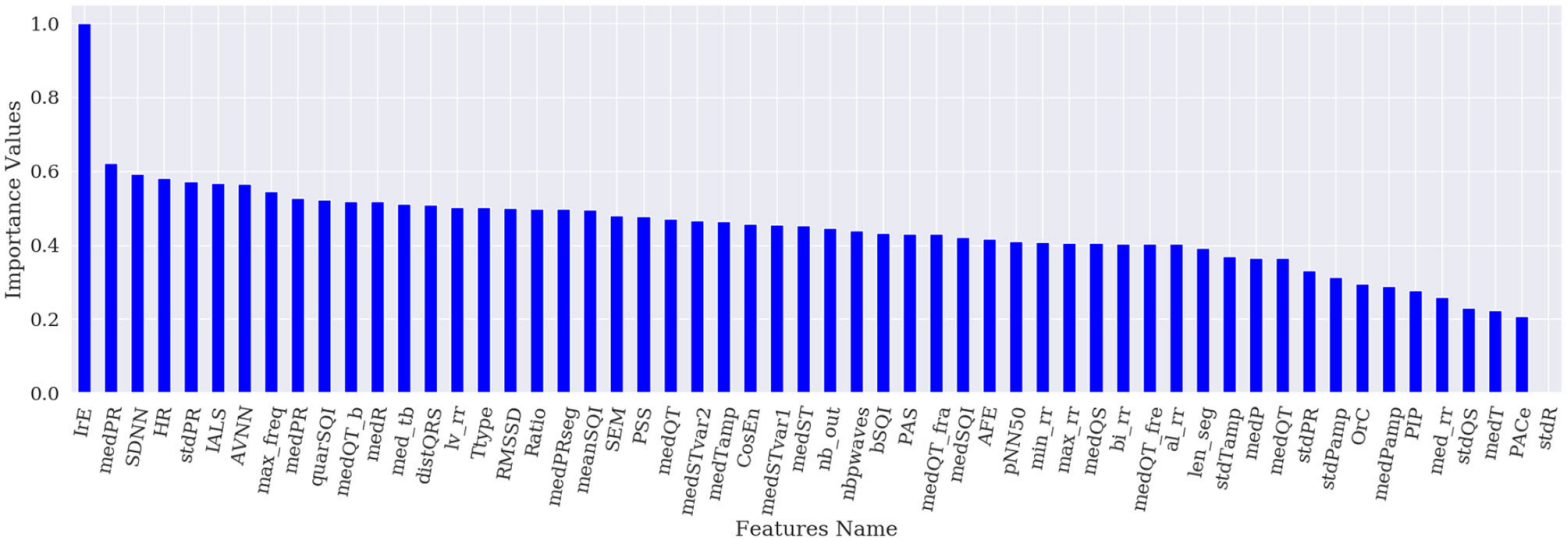
Feature importance based on LR.

**Figure 2:**
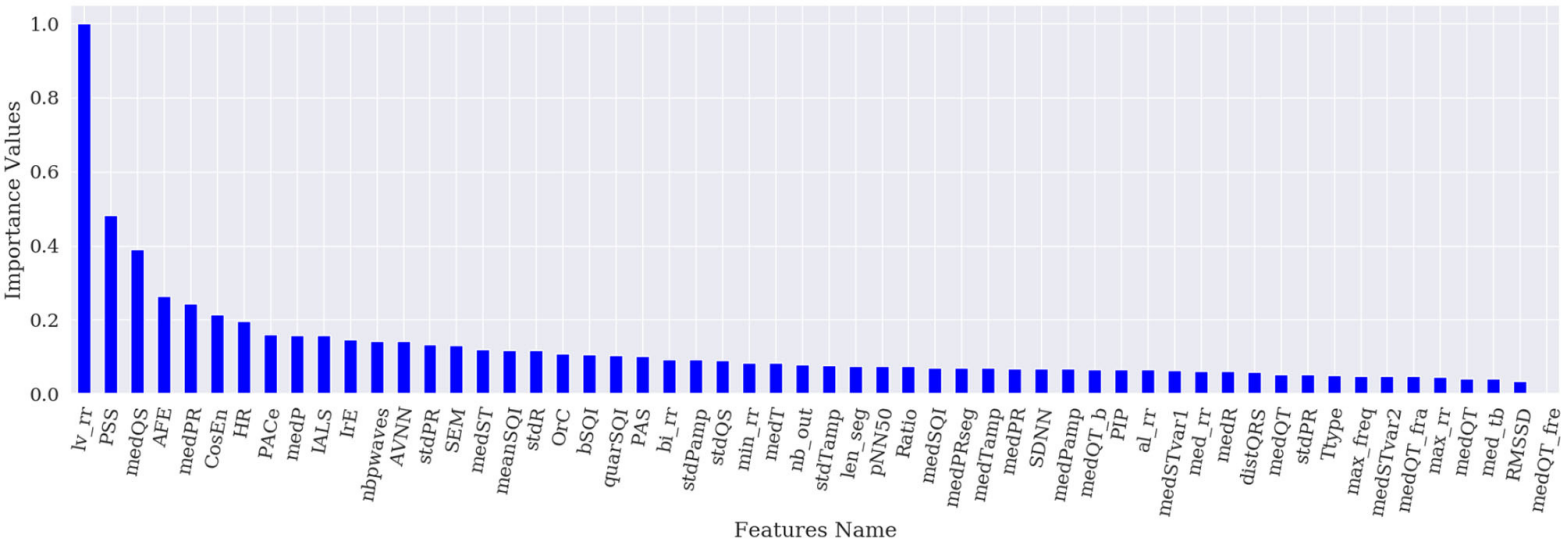
Feature importance based on PT.

**Figure 3:**
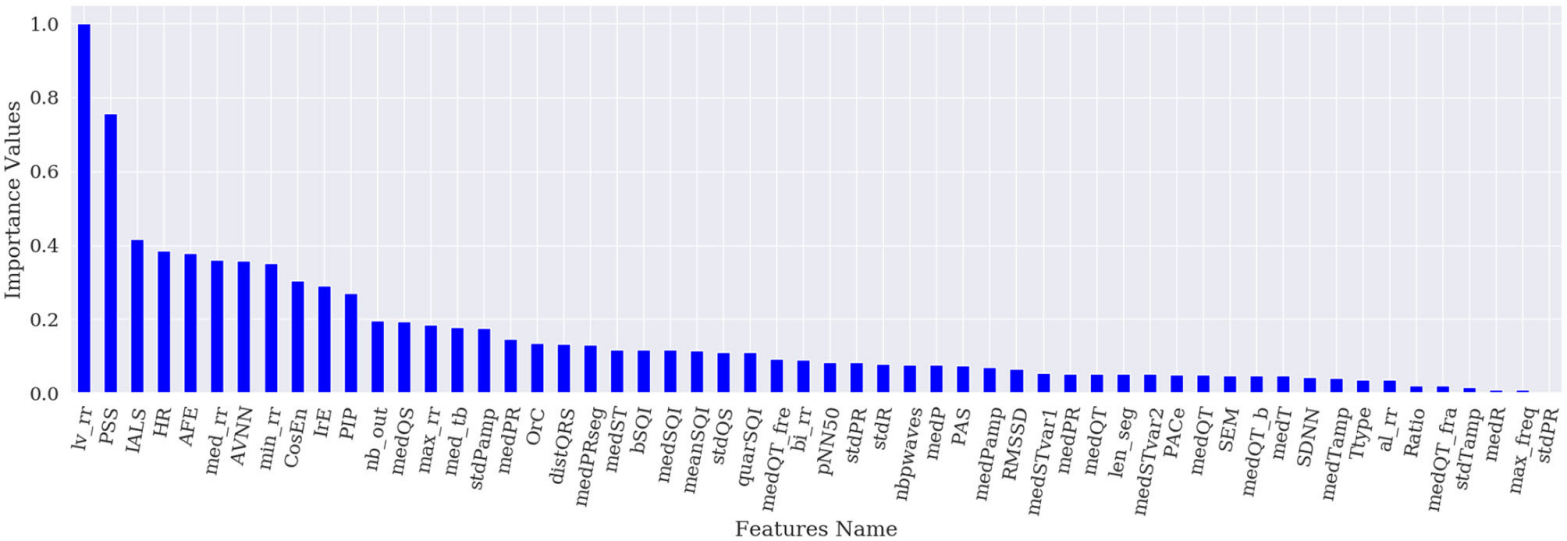
Feature importance based on RF.

**Figure 4:**
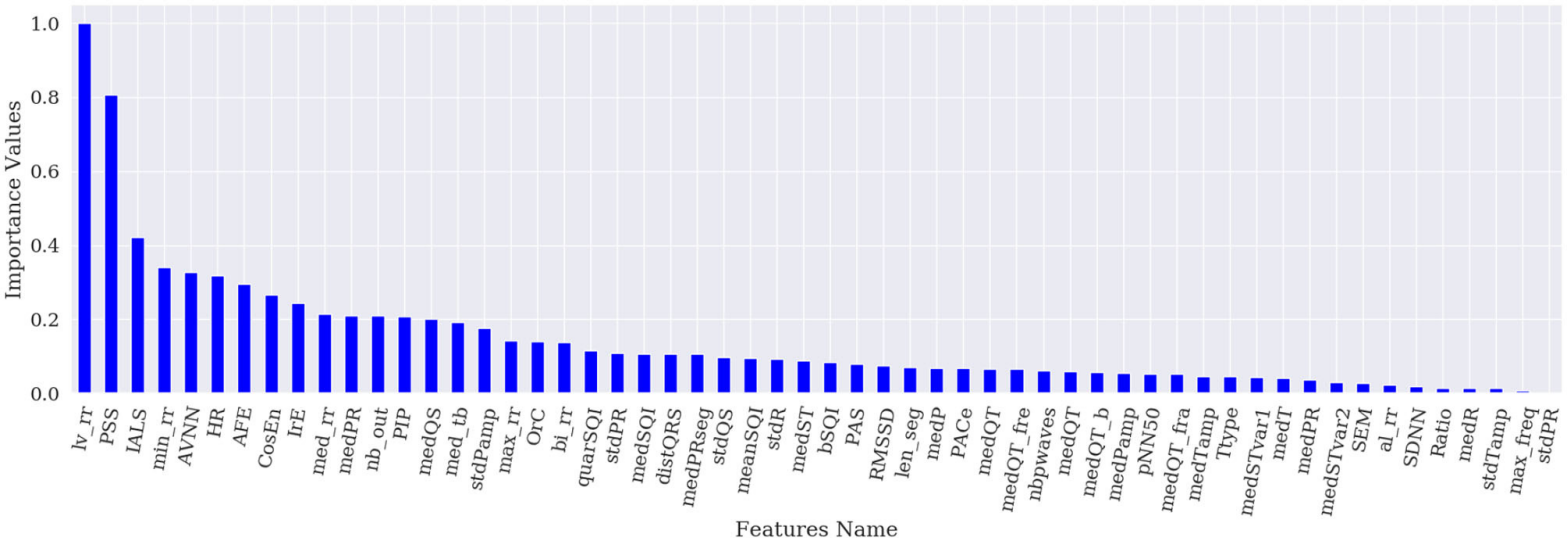
Feature importance based on SHAP.

Figures 1–4 show the feature importance for LR, PT, RF, and SHAP, respectively. The higher the values on the y-axis, representing importance values corresponding to different features on the x-axis, the more important the features are. From the figures, it can be observed that the results of different techniques vary depending on their underlying methodology. In particular, the contrast between the most important features and the others is much less pronounced with LR, whereas for the other techniques a couple of features seem to be clearly more important. PT, RF, and SHAP generate a similar ranking for the features with features like (lv_rr and PSS) being amongst the most important features for all three techniques.

**Figure 5:**
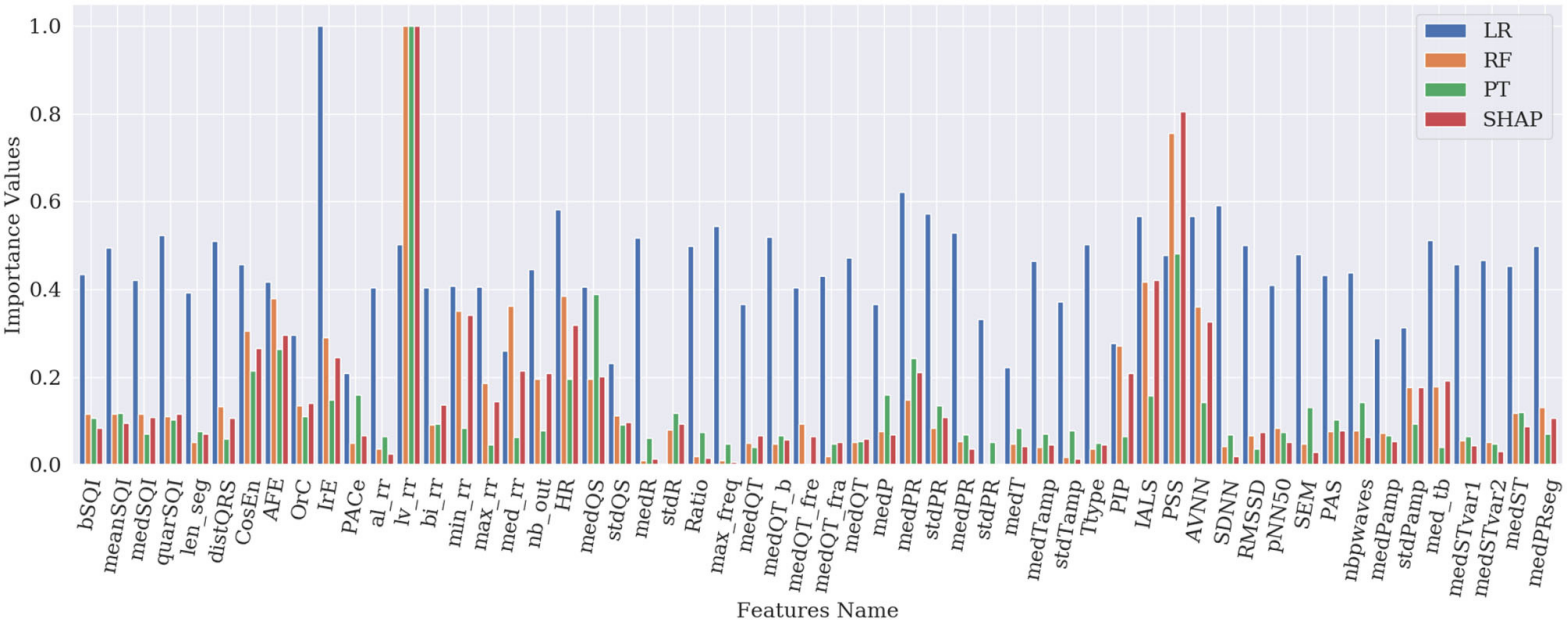
Comparison of feature importance values obtained from different techniques.

Figure 5 represents the results of the ranking of 56 features by different feature importance techniques shown in Figures 1–4 altogether. From Figure 5, the similarity between PT, RF and SHAP ranking for the extracted features can be clearly seen.

**Figure 6:**
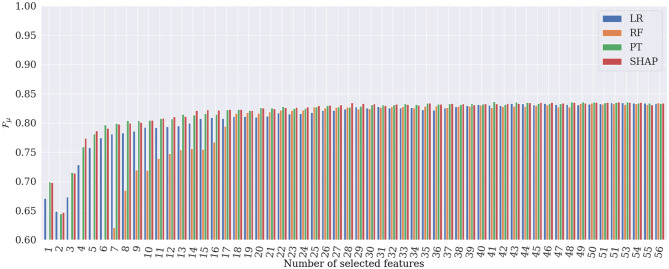
Results of heart rhythm classification in terms of $F_{\mu }$, by RF classifier applied to only the most important features. SHAP generates the best classification results based on only 28 features.

**Figure 7:**
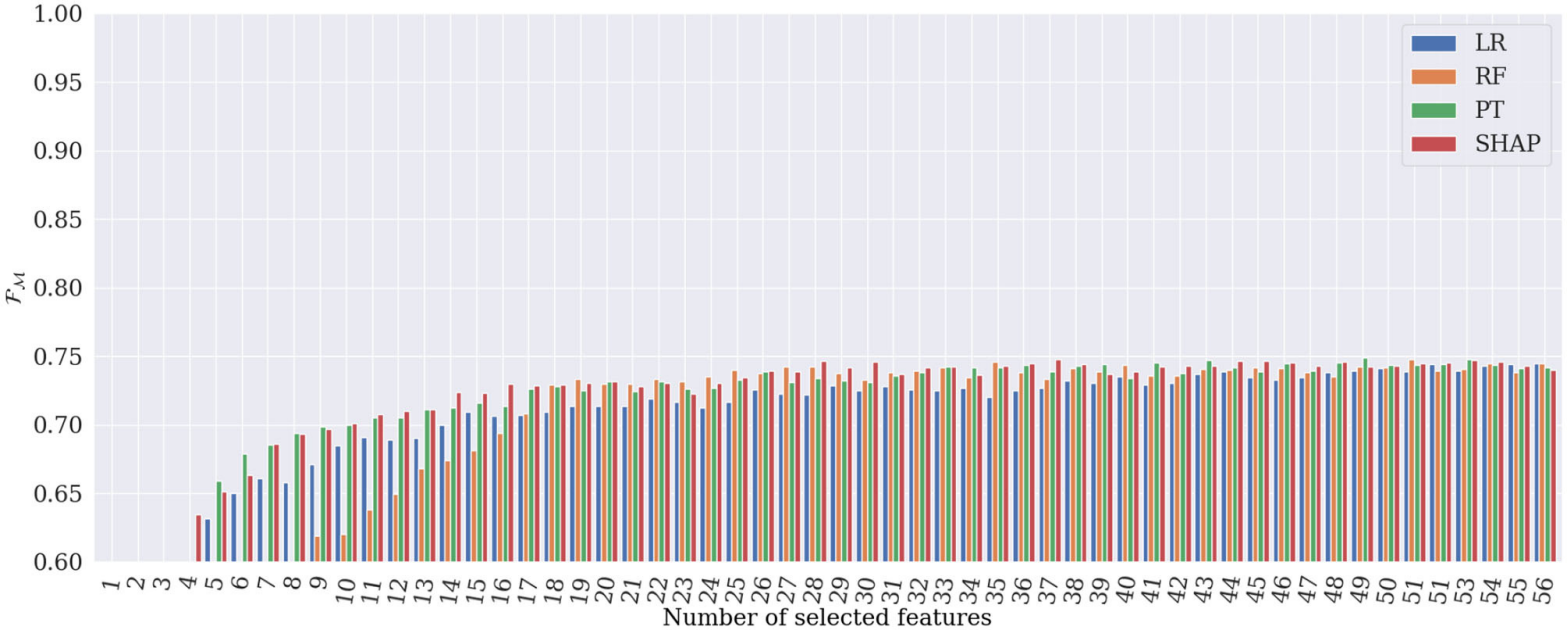
Results of heart rhythm classification in terms of $F_{M}$, by RF classifier applied to only the most important features. SHAP generates the best classification results based on only 28 features.

Figures 6, 7 show the evolution of $F_{\mu }$ and $F_{M}$ when incrementally adding features according to their importance rank for the four different global explanation techniques. According to the figures, PT and SHAP generate better results than the others (LR and RF) even when the number of features is low, with faster performance increase compared to the other two techniques. As can be expected, once all 56 features are selected, all techniques obtain almost identical $F_{\mu }$ and $F_{M}$ values. However, the subtle difference is due to the order of the presentation of features in the tree's construction in the RF classifier, which is randomly selected. It is interesting that by selecting only 28 features ranked by SHAP, the best $R_{M}$ and $F_{M}$ are achieved in the classification. This proves that the applied SHAP technique generates more reliable feature importance making the classification less complex and more computationally efficient.

**Figure 8:**
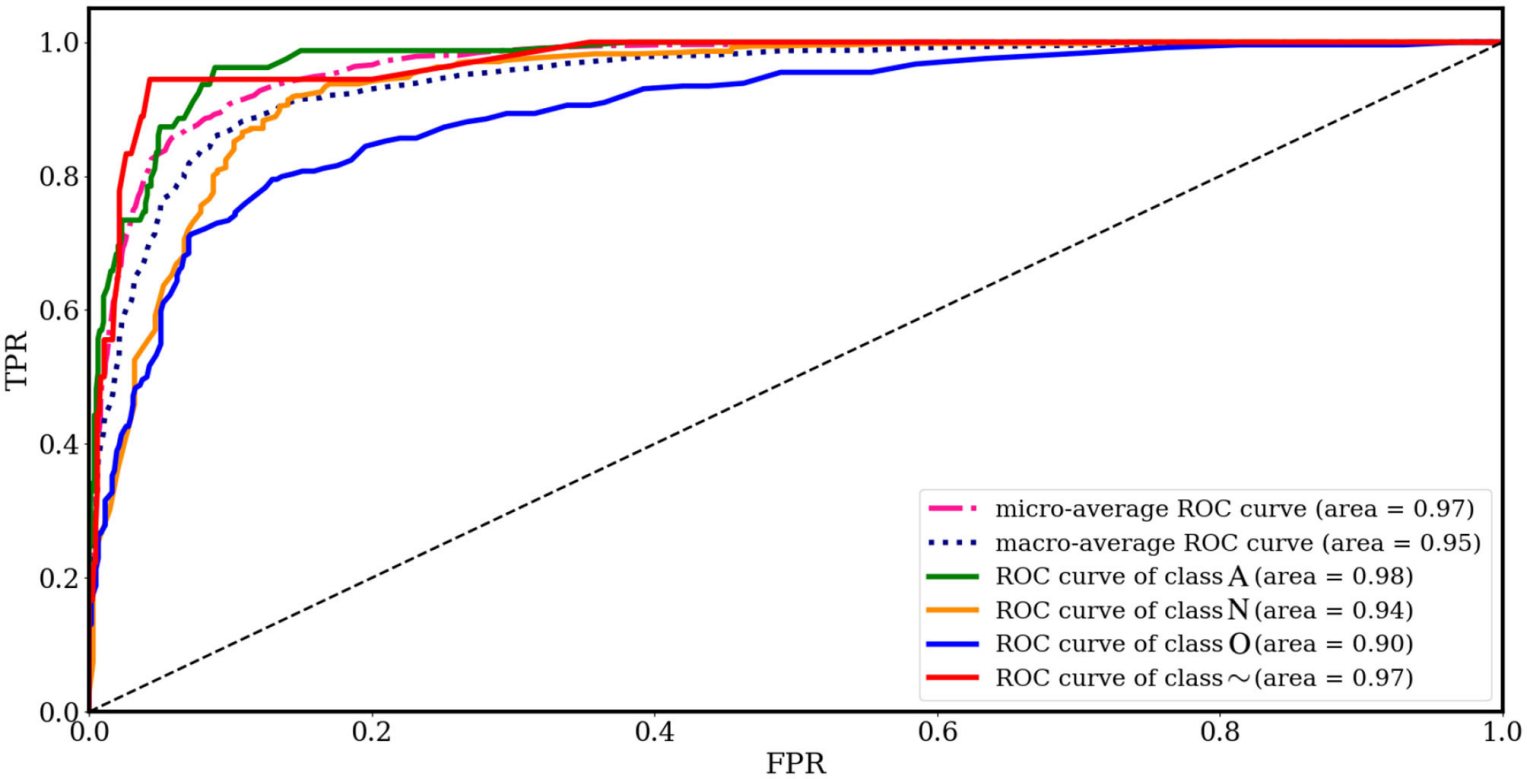
ROC curves for different classes obtained from feature importance by SHAP and RF classification and 10-fold cross-validation.

Figure 8 shows the results of RF classification based on the 28 ranked features obtained from SHAP as the best method proposed in this paper, in terms of AUC for each class, separately. Among the AUC values, the 0.98% AUC proves the effectiveness regarding AF detection. Table 3 provides the average confusion matrix obtained by the SHAP_*RF* during a 10-fold cross-validation procedure. Also, in Table 4, the results of heart rhythm classification by using ranked features and RF as the underlying classifier are presented and compared with the results of the works of Behar et al. (2017) and Pyakillya et al. (2017). The results show the effectiveness and efficiency of SHAP technique along with RF for the classification with a mean F-score of 0.746 and they also highlight how *SHAP_RF* method can provide cardiologists with a more compact set of features and tangible information in their diagnosis.

**Table 3:** Average confusion matrix obtained from feature importance by SHAP and RF classification based on 10-fold cross-validation.

	***n***	**a**	**o**	****~****
N	4,724	16	293	17
A	37	519	167	15
O	613	97	1,715	31
~	74	15	50	145

**Table 4:** Results of heart rhythm classification by using ranked features and RF as the underlying classifier.

**Method**	**#features**	**$F_{\mu }$**	**$P_{M}$**	**$R_{M}$**	**$F_{M}$**
LR_RF	52	0.834	**0.786**	0.714	0.744
PT_RF	56	0.834	**0.786**	0.714	0.744
RF_RF	41	0.836^*^	0.785	0.716	0.745
SHAP_RF	**28**	0.835	0.783	**0.720**	**0.746**
Behar et al. (2017)	35	0.730^*^	0.738	0.705^*^	0.706^*^
Pyakillya et al. (2017)	10100	0.831^*^	0.779^*^	0.713^*^	0.742

(*) *shows the non-parametric statistical difference between SHAP_RF method and the corresponding method in terms of a specific measure $F_{\mu }$, $P_{M}$, $R_{M}$, or $F_{M}$*.

Moreover, Table 5 presents a comparison between the best results generated by the techniques applied in this paper and some of the existing methods proposed by Behar et al. (2017) and Pyakillya et al. (2017), for the classification of ECG signals on CinC/PhysioNet dataset. The column *#features* depicts the number of the most important feature generated by the feature importance techniques which cause the best results in the classification. The best value of each column is highlighted in bold. To make the table readable, we use the abbreviations as *LR*_*RF*, *PT*_*RF*, *RF*_*RF*, *SHAP*_*RF*, where the name before “_” shows the name of the feature importance and model agnostic technique and the name after stands for the RF classifier. In the method proposed by Behar et al. (2017), a set of 35 features are selected based on the SVM classifiers and a CSVM classification is performed. In the method proposed by Pyakillya et al. (2017), a 1-Dimensional Convolutional Neural Network (1D-CNN), whose input is the raw ECG signal of length 10100, is applied for the feature selection and classification. The comparison with the state-of-the-art techniques shows that RF classification based on the ranked features obtained from SHAP achieves the best results for AF detection.

**Table 5:** Comparison with state-of-the-art methods on CinC/PhysioNet dataset based on cross-validation.

**Method**	**#features**	**$F_{n}$**	**$F_{a}$**	**$F_{o}$**	**$F_{p}$**	**$F_{\text{mean}}$**
LR_RF	52	0.896	0.742	0.719	0.534	0.722
PT_RF	56	0.899	0.740	0.730	0.596	0.741
RF_RF	41	0.898	0.751	0.728	0.580	0.739
SHAP_RF	**28**	0.900	0.768	0.733	0.579	0.745
Zabihi et al. (2017)	150	0.904	0.794	0.756	0.611	0.818
Datta et al. (2017)	188	0.909	0.797	0.771	-	0.826
Teijeiro et al. (2018)	–	**0.960**	**0.842**	**0.864**	**0.724**	**0.889**

In order the overall performance of the proposed compact approaches with the state-of-the-art. Measures $F_{n}$, $F_{a}$, $F_{o}$, $F_{p}$, corresponding to F1-measure for the classes N, A, O, and ~, and $F_{\text{mean}}$ as proposed in (Clifford et al., 2017) were evaluated during 10-fold cross-validation for the proposed methods and compared with the scores self-reported during cross-fold validation by the three best entries of the 2017 CinC/PhysioNet challenge (Datta et al., 2017; Zabihi et al., 2017; Teijeiro et al., 2018).

### 4.3. Local Explanation

**Figure 9:**
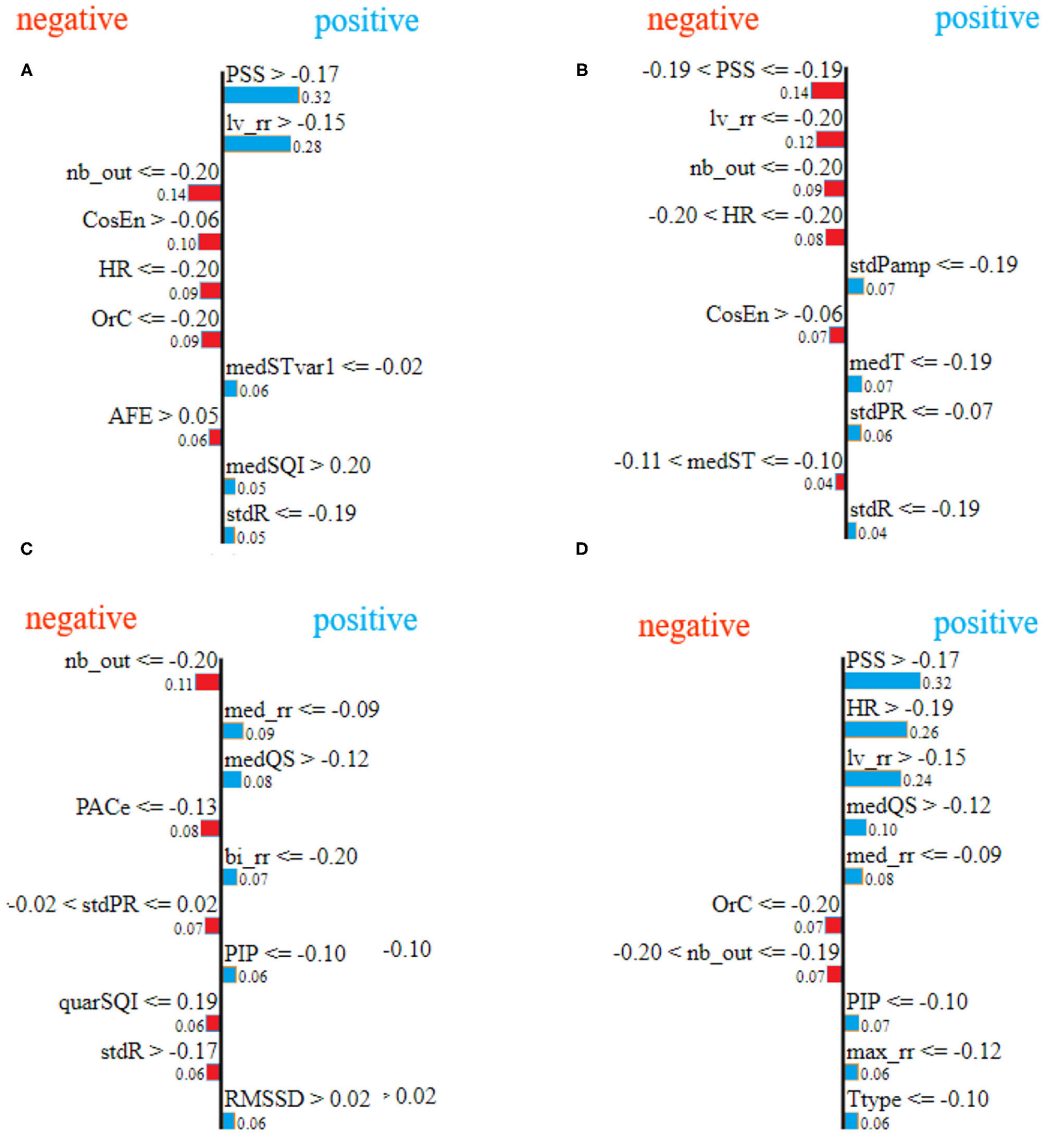
LIME feature importance for different samples, selected randomly from test set, corresponding to class atrial fibrillation **(A)**, class normal sinus rhythm **(B)**, class other rhythms **(C)**, and class noisy recordings **(D)**, respectively.

Figure 9 shows the explanation for the 10 most important features for four examples from the test set (one for each class). In Figure 9, blue indicates the features explaining the positive class prediction, while the features in red indicate the reasons why the specific sample is not classified as one belonging to the negative classes. For example, in Figure 9A, the explanation is provided for one sample from the AF class showing that the features PSS and lv_rr are the most important ones which influence the classification of the sample. Specifically, the values of the feature PSS higher than −0.17 and the values of the feature lv_rr higher than −0.15 reason that the sample belongs to class A, while the features nb_out lower than or equal to −0.20 and CosEn higher than −0.06 explain why the classifier believes the sample cannot belong to another class than AF. Finally, the low value of nb_out also explains why the classifier cannot be part of another class especially the class Other, as this feature is high in presence of multiple Premature Ventricular Contractions (PVCs) or Premature Atrial Contractions (PACs). The end-user can then understand that the AF classification was made because of the irregularity of RR intervals, high heart rate, and low prevalence of PVCs and PACs.

**Figure 10:**
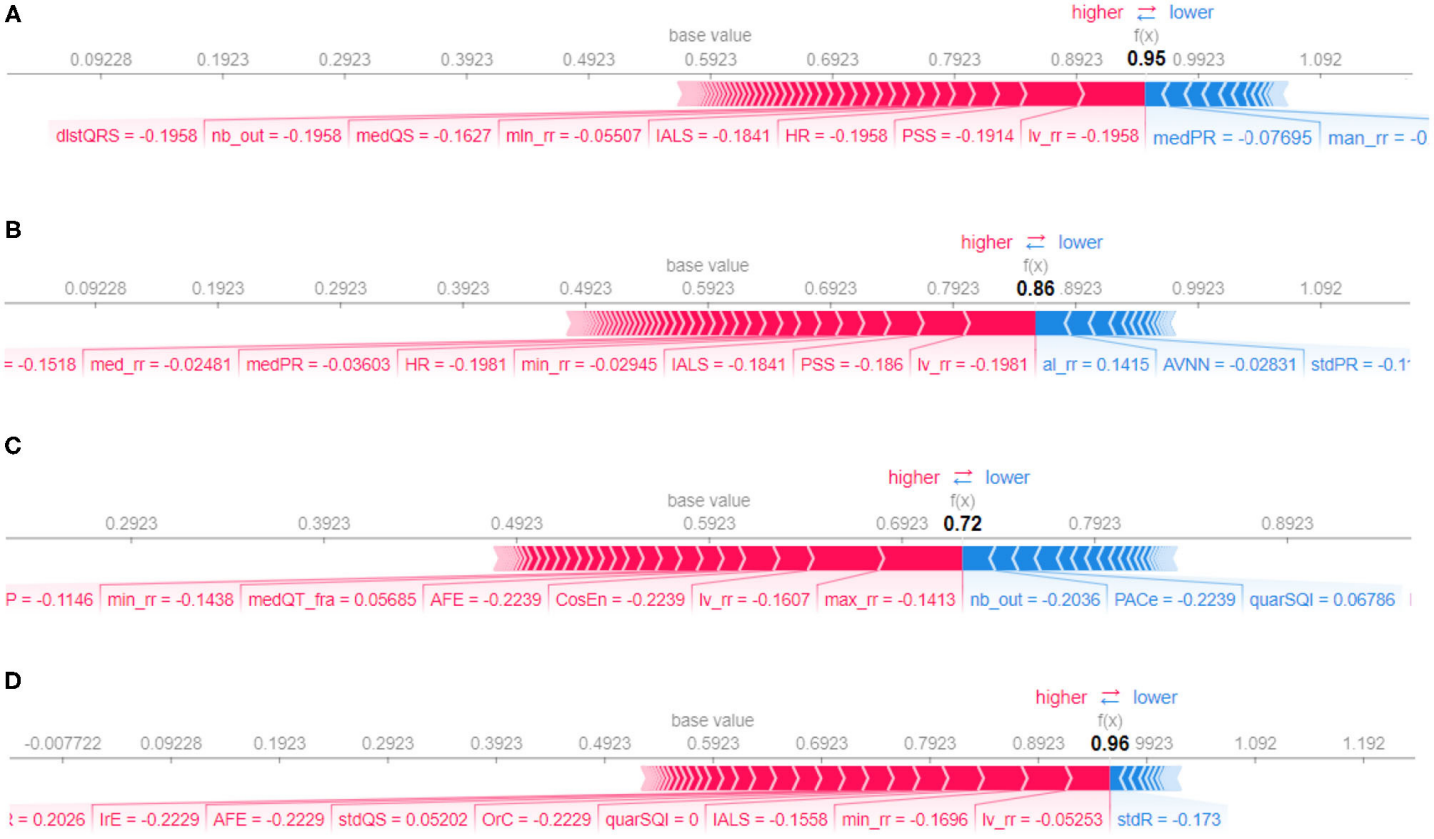
Force plot for different samples, selected randomly from test set, corresponding to class atrial fibrillation **(A)**, class normal sinus rhythm **(B)**, class other rhythms **(C)**, and class noisy recordings **(D)**, respectively.

Figure 10 represents the so-called force plots (Lundberg et al., 2018) for the same four examples used to illustrate the LIME technique in Figure 9. The arrows below the line of each plot indicate all the feature values that are moving the probability of prediction from/to the base value, which is the average model output over the training dataset. The output value, which is in bold, is the sum of the base value and the effects of the features. Features that decrease the probability of positive class are in blue and the ones that increase this probability are in red. Feature values in red move the prediction to larger values from the base value and blue arrows to smaller ones. For example, for the explanation in Figure 10A, we see that the features PSS and lv_rr move the prediction from the base value to a larger value, while the features medPR and max_rr move to smaller, resulting in the prediction probability of 0.95 for the AF class (the positive class with target value 1 in the binary classification).

By comparing Figures 9, 10, it can be seen that there is agreement on the most important features, e.g, the feature PSS and lv_rr for the same samples from atrial fibrillation and normal classes, Figures 9A,B and 10A,B, respectively. Also, Feature nb_out is the most important feature for the explanation of the sample provided in Figures 9C, 10C. There is also a discrepancy between the two techniques, e.g., feature lv_rr is the most important feature in Figure 10D, but is only estimated to be the third most important feature in Figure 9D. This can be explained by the different characteristics and assumptions of the techniques.

## 5. Discussion

The 2017 CinC/PhysioNet challenge has shown that despite advances in deep learning techniques, hand-crafted features based machine learning techniques can still achieve highly performing rhythm classification tasks. However in order to train these models, it is necessary to implement and input a large number of features (typically in the hundreds for top-performing teams). This means that given the complexity of the models, combined with non-linear classifiers (SVM, GB, and so on), interpreting the decision process is difficult. To gain the end-users (cardiologists) trust, it is essential to be able to provide an explanation of the model, and to understand how an automated decision is taken.

First, global explanation provides an interpretation of the training process and ranks the features by importance. Although global explanation is relatively complex, especially in the case of multi-class classification (since the techniques like SHAP generate importance values for different classes), end-users can understand what the model (and which features) is primarily looking at. Figure 5 shows that the model seems to be primarily interested by features based on the RR variability, lv_rr which looks at the ratio of RR intervals with large variations, HRV based features, or features based on the irregularity of the RR (e.g., IrE, AFE, CosEn, and so on). Moreover, these global explanations can be used as a feature selection technique and provide a more compact set of features and therefore less complex ML model. Among the applied techniques, SHAP seems to be working best for the explanation of RF classifier at least and provides an efficient model on the most compact set of features. The use of this most compact set of features could therefore be used and implemented on resource-constrained settings such as for mobile applications. In this study, we have focused on the initial set of features suggested by Behar et al. (2017), which contains features based on similar physiological phenomenon and can therefore be correlated. SHAP being based on collaborative game theory is well-adapted to deal with these correlated features and is able to select a compact set of features providing with good outputs. It would be interesting to analyse how SHAP values would perform on an even larger set of features.

Local explanation techniques are also interesting and complementary to global approaches, as they provide additional feedback to the cardiologists, which are specific to a given sample. We evaluated the effectiveness of the global explanation techniques by feature selection and classification, while validation of local approaches is more difficult as additional feedback is provided for each sample but does not impact the classification results. Reviewing the local explanation techniques may help the cardiologists to gain trust in the automated diagnosis, as it can confirm or infirm that the automated model is looking at a characteristic of the signal that makes clinical sense. For example, if a model focuses on high RR variability for highly artifact signals, cardiologists can discard the decision. Similarly, end-users can have more trust in a model that locally focuses on the QRS width for the detection of PVCs. Unlike, LIME which perturbs data around an individual prediction to build a model, SHAP computes all permutations globally to get local accuracy. So, LIME is faster than SHAP and it can be considered as a subset of SHAP. SHAP values can be calculated for any tree-based model. SHAP explains the prediction of the underlying model, meaning that it does not train a surrogate model, so there is no risk in having the explainer, which predicts and explains a different result. Given the review, between the LIME and SHAP techniques for a local explanation, SHAP seems to generate reliable results.

## 6. Conclusion

Machine learning has been successfully applied to improve the effectiveness of Computer-Aided Diagnosis (CADx) systems for Atrial Fibrillation (AF) detection. Providing an explanation for the decision made by CADx is considerable from cardiologists' point of view. In this paper, a range of interpretability techniques has been applied to hand-crafted features based ML models for heart rhythm classification particularly AF detection. We tested different global and local explanation feature importance techniques. We validated the impact of the techniques by applying feature selection according to the obtained feature importance and classification to the public short electrocardiography (ECG) dataset of CinC/PhysioNet. It has been shown that each feature importance technique results in different feature rankings, depending on their characteristics and assumptions. The results prove the effectiveness and efficiency of SHapley Additive exPlanations (SHAP) technique along with Random Forest (RF) for the classification of the ECG signals particularly for AF detection, as an interpretable hand-crafted feature-based model.

## Data Availability Statement

The raw data supporting the conclusions of this article will be made available by the authors, without undue reservation.

## Author Contributions

RR conducted the experiments, obtained the results, and contributed most to the writing of the paper. All authors contributed to the choice of methods, the design of the experimental protocol, and the polishing of the paper.

## Conflict of Interest

The authors declare that the research was conducted in the absence of any commercial or financial relationships that could be construed as a potential conflict of interest.
